# Time Pressure Inhibits Dynamic Advantage in the Classification of Facial Expressions of Emotion

**DOI:** 10.1371/journal.pone.0100162

**Published:** 2014-06-18

**Authors:** Zhongqing Jiang, Wenhui Li, Guillermo Recio, Ying Liu, Wenbo Luo, Doufei Zhang, Dan Sun

**Affiliations:** 1 School of Psychology, Liaoning Normal University, Dalian, China; 2 Humboldt-Universitaet zu Berlin, Berlin, Germany; Birkbeck College, United Kingdom

## Abstract

Recent studies suggest an advantage in the recognition of dynamic over static facial expressions of emotion. Here, we explored the differences in the processing of static and dynamic faces under condition of time pressure. A group of 18 participants classified static and dynamic facial expressions (angry, happy, and neutral). In order to increase the goal-directed attention, instructions emphasized speed and announced time pressure in the interval for the response (maximal 600 ms). Participants responded faster and more accurately in the static than in the dynamic condition. Event-related potentials (ERPs) showed larger amplitude of the P1 (90–130 ms) and LPC (300–600 ms) components for dynamic relative to static stimuli, indicating enhanced early visual processing and emotional attention. On the other hand, the N170 was more negative in static relative to dynamic faces, suggesting better structural encoding for static faces under time pressure. The present study shows some advantages in the processing of static over dynamic facial expressions of emotion when the top-down (goal-driven) attention is strengthened.

## Introduction

The recognition of facial expressions is fundamental in human communication. Most of the numerous studies investigating the recognition of facial expressions of emotion recognition used static images of faces as stimuli [1]. However, in real life people usually confront dynamic facial expressions with rapid changes.

Recently, some studies compared the processing of dynamic versus static facial expressions of emotions, and showed an advantage in the recognition of dynamic facial expressions. More particularly, dynamic facial expressions were recognized more accurately [2], [3], more quickly [4], and perceived with greater arousal and intensity [5], [6] than static ones. One interesting aspect of this phenomenon, so-called *dynamic advantage,* is that in static stimuli the configuration of facial muscles providing the information necessary to identify the expressions is available earlier than in dynamic. Static facial expressions show the full-intense expression already from the onset of the stimuli, whereas in dynamic stimuli, the facial expressions emerge from a neutral face, developing in several milliseconds. Hence, dynamic expressions provide some form of information that is absent in static and compensates the weak intensity of the expressions during their rising [2].

It should be noted that the dynamic advantage reported in some of those studies were not presented in all emotional categories [2]–[4]. One study showed slower processing for dynamic than static expressions, and explained this *static advantage* in terms of ceiling effects, since the static condition (i.e., the pictures of fully developed, intense facial expressions) yielded almost perfect identification [7]. Besides the perfect identification in the static, one more important factor to reverse the *dynamic advantage* is that the trials were self-paced, with each trial starting after recording the previous response. Such a design could facilitate participants focusing on task-relevant factors [8], [9], and then help the static facial expressions to get some advantage in facial categorization because they are fully developed at the onset. In contrast, in dynamic face-stimuli, the expression in the first frame after the onset is still incomplete. Considering these factors, a reduction or even an inversion of the processing advantage for dynamic over static facial expressions seems plausible, at least under some circumstances.

It is interesting that time pressure might also play similar role as the self-paced procedure on participants’ performance. Karau and Kelly [10] proposed the attentional focus model (AFM) to explain the effect of time pressure. They argue that under conditions of time pressure, temporal constraints and task demands become more salient, leading participants to focus on task accomplishment, and in turn, to attend more readily to those features that seem most important to complete the task. AFM was supported by many studies [11]–[13]. It should be noted that time pressure facilitates faster but also premature actions, as increases the number of commission errors [14].

ERP studies have shown an enhanced visual processing of emotional relative to neutral faces at different stages of processing. The P1 is a positive peak around 100 ms post-stimulus onset, maximal over posterior sites, considered to reflect the processing of the low-level features in the extrastriate visual cortex [15]. The P1 amplitude is sensitive to manipulations of attention [16], [17], and in some studies to facial expressions of emotion [18]–[20].

The N170 is negative-going deflection, peaking around 170 ms over the lateral occipito-temporal electrode sites, greater amplitude for faces compared with other stimuli, and therefore related with the structural encoding of the face [21], [22]. Like for the P1, there are inconsistent findings about the emotion effects on the N170. Some studies reported effect of facial expressions on the N170 [18], [23], [24], whereas others did not [25], [26]. Interestingly, the temporal properties of the dynamic facial expressions, like the speed in the rising time also impact the emotional modulation of the N170 [27].

The processing of facial expressions appears more consistently in ERPs after 200 ms, Subtractive-waves emotional minus neutral typically show more negative amplitudes for emotional than neutral stimuli at posterior electrodes in the so-called early posterior negativity (EPN) [28], and a positive counterpart on frontal sites [29]. The EPN is considered to reflect enhanced sensory processing of emotional stimuli [30], [31].

The late positive complex (LPC, also called late positive potential, LPP) appears as a positive going deflection over central-parietal electrodes in the time window of 300–600 ms after stimuli onset. The amplitude of LPC is thought to reflect the process of categorization and attention to motivationally relevant information [25], [32]. LPC amplitudes are normally larger for emotional stimuli of negative valence, for example, one study [27] revealed larger LPC amplitudes for dynamic expressions of anger, disgust, fear, sadness and surprise, relative to both neutral and positive (happiness) expressions, indicating higher intrinsic relevance and more attention for the processing of negative expressions at later stages.

The EPN and LPC components are larger for dynamic than static facial expressions, suggesting that the dynamic advantage relates to a gain in the early visual processing and greater allocation of emotional attention [4], [27].

According to the hypothesis of three stages in the processing of facial expressions [33], the P1 reflects a first stage of automatic processing of negative expressions; the N170 and EPN reflect a second stage, in which emotional facial expressions are distinguished from neutral; and the LPC reflects a third stage, in which emotional facial expressions are differentiated from each other.

The present study investigated the impact of time pressure in the processing of static and dynamic facial expressions of emotion. We hypothesize that introducing time pressure in the emotion classification task will encourage participants to focus in the early interval after stimulus onset to extract emotional information as soon as possible, in which dynamic expressions are incomplete as they are still emerging from neutral. Therefore, static emotional expressions may be processed more accurately and quickly than the dynamic. To test this hypothesis, the present study used a similar procedure as [4], and introduced time pressure by setting a limited time window for the stimuli presentation and the participants’ response.

We expect a reduction or even an inversion of the dynamic advantage, and hence, faster reaction times (RTs) for the classification of static than dynamic expressions. Congruently, the effect of facial expressions (happy, neutral, and angry) in the ERPs might appear earlier in static than dynamic stimuli. Regarding the effect of presentation mode (dynamic, static) in the ERPs, we have two alternative hypotheses. The first hypothesis predicts that the dynamic advantage will emerge in ERPs earlier than the EPN, as shown in [4] where there was no time pressure to respond. The effect of presentation mode might reflect the advantage of dynamic faces grabbing attention and the processing of more complex information (i.e., multiple frames, motion, and expressional change) than the static. The second hypothesis predicts some form of *static advantage* in the ERPs reflecting a better processing of static stimuli due to its full and constant expression, compared with the changeable and incomplete expression in the dynamic stimuli.

## Method

### Participants

A group of 20 participants (five men; *M* = 25.05 years old, *SD* = 1.05, range = 22∼27) rated the face-stimuli in terms of valence that were used afterwards in the main experiment. Another group of 18 participants (eight men; *M* = 23.7 years, *SD* = 1.21, range = 21∼27 years) attended the main experiment. All participants had normal or corrected-to-normal vision and reported no history of brain injuries, medications or drugs consumption. Participants were students at Liaoning Normal University and gave informed written consent prior to participation in the studies. All participants received a gift after the experiment. The ethical review committee of Liaoning Normal University approved the protocol.

### Materials

All stimuli were created with the animation software FaceGen [34], which can create face pictures and allows control of emotional intensity, gender and ethnicity in an analog scale. The numbers of face stimuli of each ethnicity and gender are shown in Table 1. Crosstabs test revealed the numbers of male and female photos did not differ significantly among different treatments (race by facial expression), for male photos, *χ* ^2^ = 1.20, *df* = 2, *p* = 0.55; for female photos, *χ* ^2^ = 0.36, *df* = 2, *p* = 0.84.

**Table 1:** The number of face stimuli of each ethnicity and gender.

	Male			Female		
	Angry	Happy	Neutral	Angry	Happy	Neutral
Western	13	7	13	10	15	13
Asian	9	8	7	8	10	7

In the static condition, stimuli consisted of face images showing happy, angry (fully-intense) or neutral facial expressions. In the dynamic condition, emotional expressions were displayed in a series of three consecutive pictures progressively increasing the intensity of the expression. The expression intensity of the first picture was 33%; the second was 66%; and the third showed the point of maximal intensity (100%) displayed also in the static condition. The first and second pictures were presented for 50 ms each. The third picture showing the fully-intense expression was displayed frozen until participants responded, with a maximal presentation time of 500 ms. For the three pictures in the dynamic neutral expressions, the first and the third pictures showed static neutral expression with open eyes, and the second image showed the face with closed eyes, giving the impression of a blink. Hence, three facial expressions (happy, angry, and neutral) were displayed in static and dynamic fashion. Each of the static and the dynamic stimuli was maximally presented for 600 ms. Exterior features (i.e., hair, ears, and neck) were covered with an oval shape.

In the pre-rating study, participants rated the emotional valence of the static stimuli, namely, the fully intense emotional expressions and neutral. Ratings were performed on a 9-point scale (1 = *most negative*, 9 = *most positive*). Results differed among angry (*M* = 2.40, *SD* = .53), neutral (*M* = 4.37, *SD* = .59), and happy (*M* = 6.75, *SD* = .75). ANOVA revealed a significant main effect of valence, *F* (2, 57) = 81.28, *p*<.001. Post-hoc multiple comparisons showed that all pairs were significant (all *p*s<.01): angry vs. happy, *t* (38) = 21.18; happy vs. neutral, *t* (38) = 11.16; angry vs. neutral, *t* (38) = 11.00.

### Procedure

**Figure 1:**
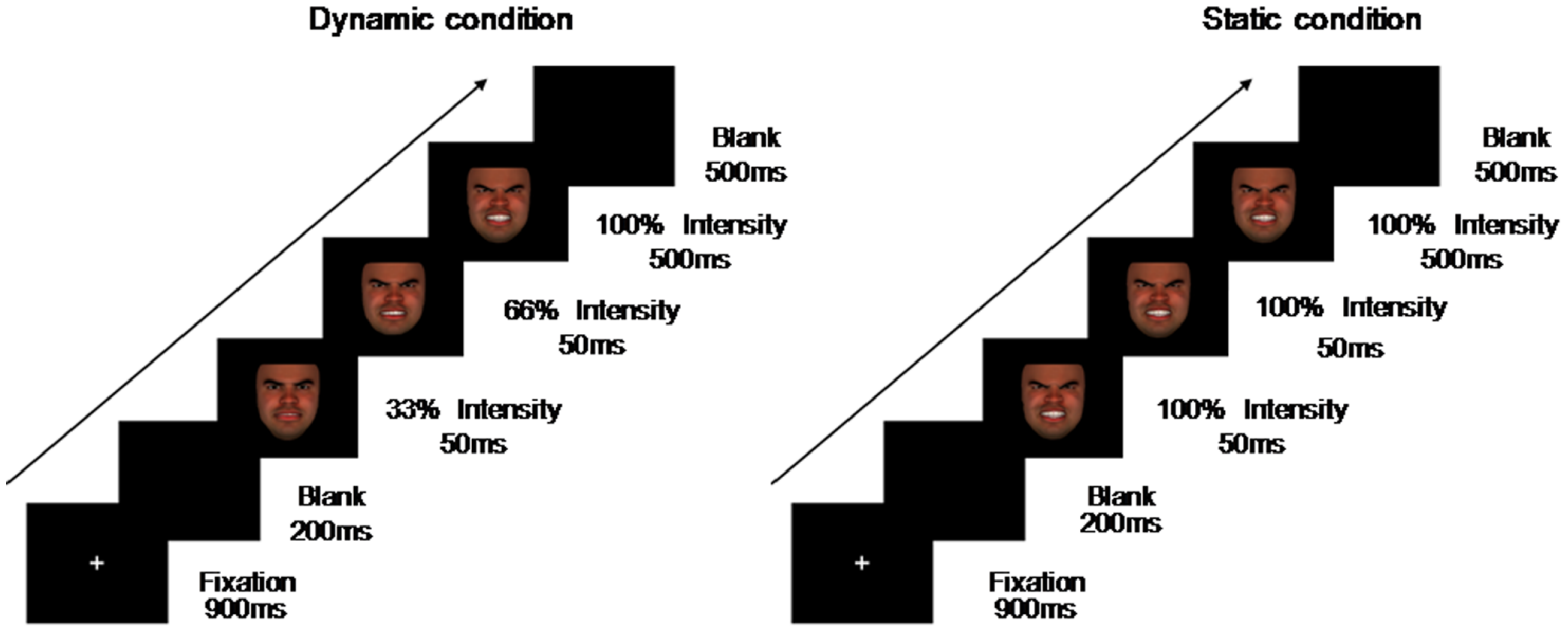
The sequence of events in a trial showing emotional facial expressions of anger in static (right) and dynamic (left) presentation modes.

Participants seated in a comfortable chair in a dimly illuminated room at one meter of distance from a computer screen. Stimuli were presented using E-prime v1.2. Figure 1 shows the sequence of events in trials of static and dynamic conditions. Participants were instructed to classify the facial expressions as angry, happy, or neutral as soon as possible after the stimuli appeared but before it disappeared (max. 600 ms) by pressing one of three keys with the index and middle fingers. The response pattern was counter-balanced among participants. Pictures disappeared when participants gave a response. If no response was given within the 600 ms that the stimuli were presented, the stimulus disappeared. The whole experiment included six practice trials and 240 experimental trials (each condition had 40 trials). Feedback for correct and incorrect responses was provided in practice trials only. Participants started with the experiment only after their accuracy rate (ACC) in the practice trials achieved 100%. Short, self-administered breaks appeared after 60 trials. The sequence of trials was fully randomized across participants.

### Data Collection

Behavioral data included RTs and ACC. RTs were calculated from the onset of the stimuli (in the dynamic condition this point was the onset of the first picture of the sequence). All trials with RTs slower than 600 ms were marked as missed and discarded from further statistical analyses in behavioral and ERP data (18.85% of the total).

The electroencephalogram (EEG) was recorded with a 128-channel EEG system (EGI, Eugene, USA) and filtered online using 0.1–100 Hz band-pass filter at 250 Hz sampling rate with a 22 bit A/D converter. The vertex channel (Cz) was used as reference during acquisition. Electrode impedances were kept below 50 kΩ. According to Ferree, Luu, Russell, and Tucker [35] recommendations for EGI EEG system, there are no significant changes in amplitude in any EEG frequency bands by increasing the scalp-electrode impedance from less than 10 kΩ to (≈) 40 kΩ (most about 50 kΩ). Offline, the continuous EEG data was filtered with a 30 Hz low-pass, re-calculated to average reference and then segmented relative to the onset of the first facial expression picture (200 ms before and 600 ms after) according the experimental conditions. After averaging, segments were baseline corrected 200 ms before stimulus onset.

Segments including eye blinks, eye movements or other artifacts were excluded from analyses (5.01% of trials with correct responses). Segments were considered with artifacts if they contained ten or more channels exceeding a voltage threshold of 200 µv (absolute) or a transition threshold of 100 µv (sample to sample). Statistical analyses were conducted over 76.92% of total trials. The numbers (*M*±*SD*) of trials in the dynamic condition are: angry (31±5), happy (30±5) and neutral (29±4); and in the static: angry (33±4), happy (32±4), and neutral (30±5).

## Results

Repeated measures analyses of variance (ANOVA) were conducted on behavioral and ERP data with SPSS11.5 (SPSS Inc., Chicago, IL, USA), with the factors presentation mode (2 levels: dynamic, static), and facial expression (3 levels: happy, neutral, angry). Analyses of the ERP data included also the factor of electrodes cluster position (2 levels: left, right). *F* values and degrees of freedom were Greenhouse-Geisser corrected when the variance sphericity assumption was not satisfied. Post-hoc tests were corrected for multiple testing using the Bonferroni correction. The partial eta-squared value (*η_p_^2^*) indicate estimates of the effect size.

### Behavioral Results

Mean RTs and ACC are presented in *Table* 2. For RTs, the ANOVA showed a main effect of presentation mode, *F* (1, 17) = 23.66, *p*<.001, *η_p_^2^* = .58, reflecting significantly slower RTs for dynamic than static condition. The effect of facial expression was not significant (*p* = .15). The interaction effect between presentation mode and facial expression was significant, *F* (2, 34) = 6.29, *p*<.01, *η_p_^2^* = .27, reflecting that the effect of presentation mode was significant for angry, *F* (1, 17) = 35.27, *p*<.001, *η_p_^2^* = .68; and happy conditions, *F* (1, 17) = 15.82, *p*<.01, *η_p_^2^* = .48, but not for neutral (*p* = .98).

**Table 2:** RT (ms) and ACC (proportion of correct trials) separated for conditions.

	Angry (*M*±*MSe*)		Happy (*M*±*MSe*)		Neutral (*M*±*MSe*)	
	RT	ACC	RT	ACC	RT	ACC
Dynamic	486.65±8.97	.81±.03	483.07±5.89	.80±.03	488.97±7.42	.77±.02
Static	465.58±7.85	.87±.02	468.01±5.88	.83±.02	488.84±5.35	.79±.03

Data from ACC showed a main effect of presentation mode, *F* (1, 17) = 18.12, *p*<.01, *η_p_^2^* = .52, reflecting lower ACC in the dynamic relative to the static condition. The effect of facial expression yielded a trend, *F* (2, 34) = 2.58, *p* = .09, *η_p_^2^* = .13. The two-way interaction was not significant (*p* = .26).

### ERP Results

**Figure 2:**
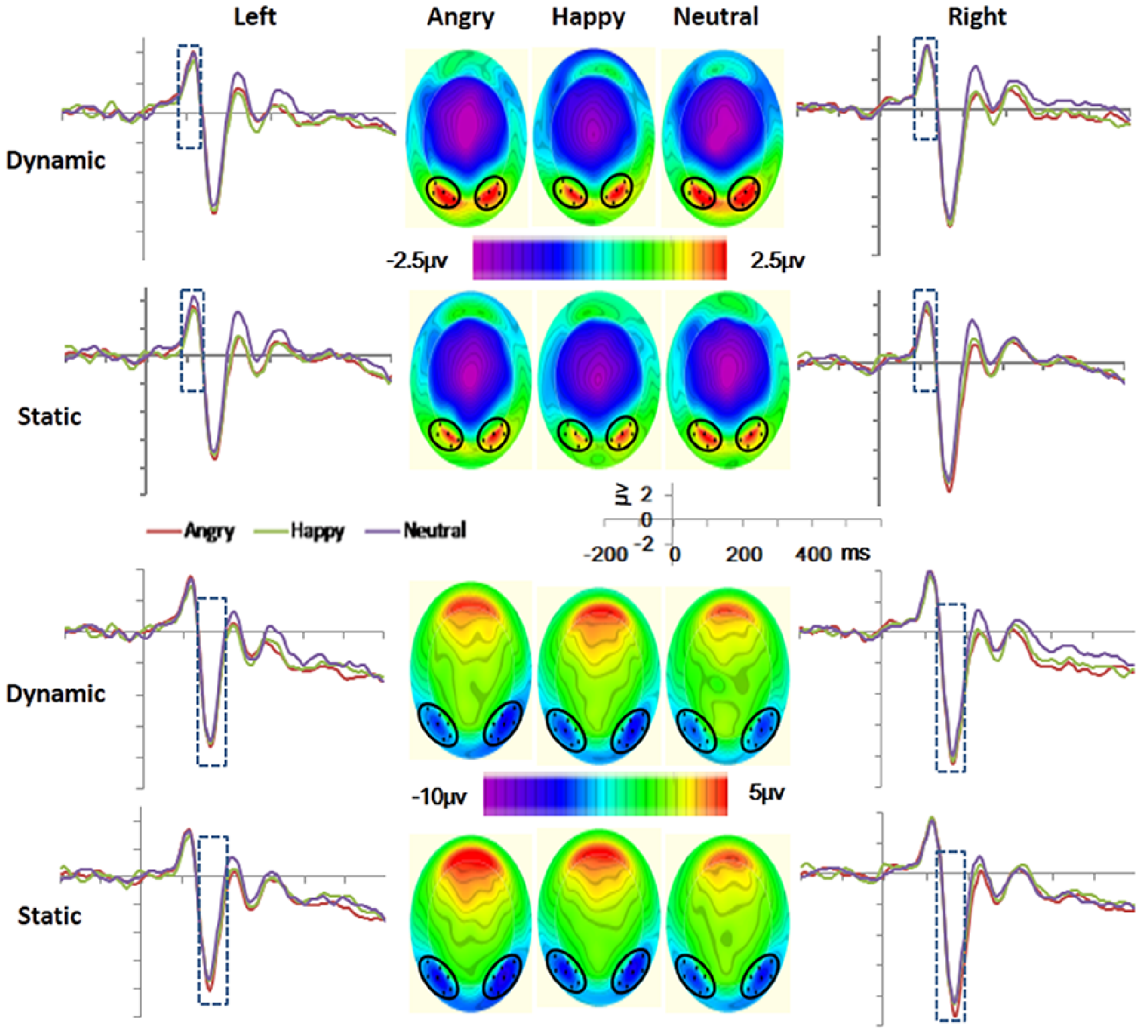
Waveforms and topographic maps (with nose up) for the P1 (top), and the N170 (bottom), showing grand averaged amplitudes at selected clusters of sensors.

**Figure 3:**
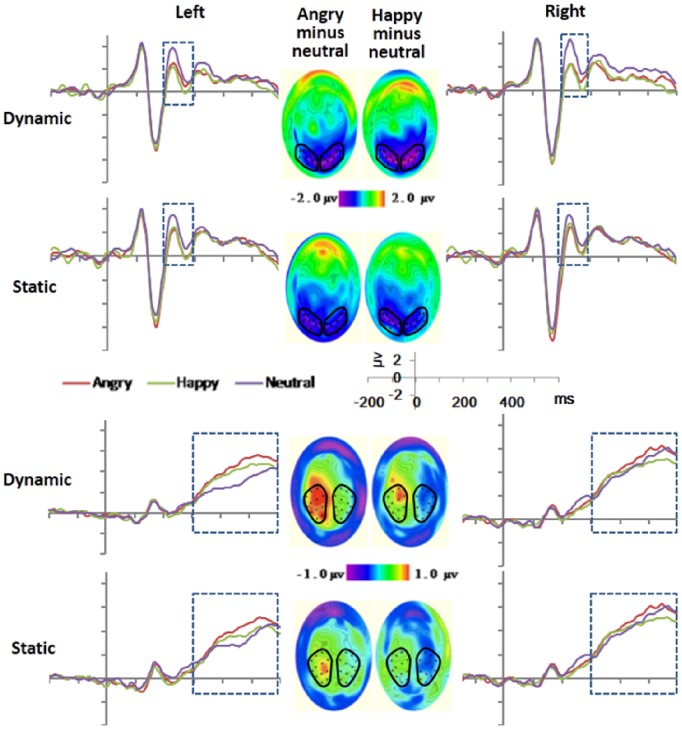
Waveforms and topographic maps (with nose up) for the EPN (top), and the LPC (bottom), showing grand averaged amplitude at the selected sensors clusters. The topographic maps of the EPN and LPC show subtractive waves of emotional (angry, happy) minus neutral.

Based on visual inspection of the ERPs (see Figure 2) and previous studies [4], [16], [17], [21], [24], two clusters (left and right) of electrodes were selected for each component (the names of the equivalent electrodes in the standard 10–20 system are also given for comparison). For the P1 the left group of electrodes includes 58 (T5), 64, 65, 69 and 70 (O1), and the right includes 83 (O2), 89, 90, 95 and 96 (T6). For the N170, the left group includes 57, 58 (T5), 63, 64, 65, 68 and 69, and the right one 89, 90, 94, 95, 96 (T6), 99, and 100. For the EPN, the left cluster includes 58 (T5), 59, 64, 65, 66, 69, 70 (O1), 71, and 74, and the right one 76, 82, 83 (O2), 84, 89, 90, 91, 95, and 96 (T6). For the LPC, the left sites were 30, 31, 36 (C3), 37, 41, 42, 47, 52 (P3), 53, 54, 60, and 61, and the right ones were 78, 79, 80, 85, 86, 87, 92 (P4), 93, 98, 103, 104 (C4), and 105. Clusters and electrodes are marked with black dots and circles in Figures 2 and 3.

Averaged ERP amplitudes were exported for each component in the selected time intervals indicated below separated for the left and the right cluster of electrodes (Figure 2).

#### P1 (90–130 ms)

Figure 2 (top) shows the waveforms and topographic maps for the P1. The ANOVA over P1 amplitudes only showed significant effect of presentation mode, *F* (1, 17) = 8.61, *p*<.05, *η_p_^2^* = .26. The P1 was larger in the dynamic (*M* = 3.36 µv, *MSe* = .44) than the static condition (*M* = 2.96 µv, *MSe* = .47). All the interactions effects and the main effects of electrodes cluster position, and facial expression were not significant (*ps*>.05).

#### N170 (130–200 ms)

For the N170 amplitude (Figure 2, bottom), the ANOVA yielded significant effects of facial expressions, *F* (2, 34) = 4. 84, *p*<.05, *η_p_^2^* = . 22 and presentation mode, *F* (1, 17) = 13.23, *p*<.01, *η_p_^2^* = .44. The two-way interaction was not significant (*p*>.05). Post hoc analysis revealed larger N170 for angry (*M = *− 4.61 µv, *MSe* = .65) than neutral faces (*M* = − 4.05 µv, *MSe* = .64), *F* (1, 17) = 7.53, *p*<.05, *η_p_^2^* = .31. The main effect of presentation mode reflected larger N170 amplitude for static (*M* = − 4.60 µv, *MSe* = .68) compared with dynamic facial expressions (*M* = − 4.13 µv, *MSe* = .63). The main effect of electrodes cluster position and its involved interaction effects were not significant (*p*>.05).

#### Supplementary analysis of P1 and N170

Since dynamic faces elicited greater P1, but less N170 than static ones, it is reasonable to doubt whether the effect of presentation mode in the N170 was simply a carry-over effect. Carry-over effects could affect the effect of presentation mode across facial expressions, but cannot account for the effect of facial expressions within each presentation mode. Hence, if effects of facial expressions appear within the static but not within the dynamic condition, this would indicate a better configural processing of some facial expressions in the static condition. To verify this question, we added statistical analysis of the facial expression effect on P1 and N170 separately for the dynamic and the static. For the P1, the effects of facial expression were not significant in the static or in the dynamic condition (*F*s<1). As expected, for the N170 the effect of facial expression was significant in the static condition, *F*(2, 34) = 4. 24, *p*<.05, *η_p_^2^* = .19, but not in the dynamic, *F*(2, 34) = 1. 38, *p* = .27. Follow-up analysis revealed a significant (*p*<.05) difference in N170 amplitude between angry static (*M = *− 4.92 µv, *MSe* = .68) and neutral static (*M = *−4.25 µv, *MSe = *.68).

#### EPN (200–300 ms)

For the EPN amplitude (Figure 3, top), we only observed a significant effect of facial expressions, *F* (2, 34) = 11. 39, *p*<.01, *η_p_^2^* = .40. EPN amplitudes were larger for angry (*M = *1.69 µv, *MSe = *.75) than for neutral (*M = *2.69 µv, *MSe = .76*), *F* (1, 17) = 13.26, *p*<.01, *η_p_^2^* = .44. EPN amplitudes were also larger for happy (*M = *1.54 µv, *MSe = *.76) expressions in relation to neutral, *F* (1, 17) = 19.25, *p*<.01, *η_p_^2^* = .53. All the interaction effects and the main effects of presentation mode, and electrodes cluster position were not significant (*p*>.05).

#### LPC (300–600 ms)

For the LPC component (Figure 3, bottom) we obtained significant main effects of facial expressions, *F* (2, 34) = 6. 51, *p*<.01, *η_p_^2^* = .28, and presentation mode, *F* (1, 17) = 7.96, *p*<.05, *η_p_^2^* = .32. Overall, the LPC was more positive in the dynamic (*M = *5.52 µv, *MSe* = .54) than in the static condition (*M = *5.13 µv, *MSe* = .51) condition. The two-way interaction was not significant. Post-hoc analyses revealed a significant difference (*p*<.05) for the comparison angry (*M = 5*.84 µv, *MSe* = .56) versus neutral (*M = *4.91 µv, *MSe* = .56), *F* (1, 17) = 11.98, *p*<.01, *η_p_^2^* = .41. The main effect of electrodes cluster position and its involved interaction effects were not significant (*p*>.05).

## Discussion

The present study investigated whether the *dynamic advantage* in the recognition of facial expressions of emotion can be inhibited by setting a limited time window for the participants to respond, thus, increasing their attention to the task [10], [13], and especially to the early time window in which the dynamic expressions are still emerging. In line with our hypothesis, we did not observe a dynamic advantage under conditions of time pressure, in fact, participants classified faster and more accurately static than dynamic expressions. Effects of presentation mode on the ERPs started as early as 100 ms after stimulus onset, as larger P1 for dynamic than static, and continued, but reversed, in the N170, which was larger for static than dynamic expressions. Effects of facial expressions appeared on the N170 time window, as larger N170 amplitude for angry than neutral, and continued during the EPN interval for both angry and happy, and during the LPC for angry. Overall results indicate a different time course of presentation mode and facial expression effects, compared with similar studies without time pressure [4].

Consistent with our hypothesis, results confirm that restricting the time window for the participants to respond can reverse the *dynamic advantage*. This finding is consistent with [7], in which the static and the dynamic stimuli were presented in separated blocks and self-paced trials. The limited response time window in our study might have enhanced the goal-directed attention, and generated an urge to extract the emotional information as quickly as possible, directing attention to the information displayed in the first 100 ms after stimulus onset, where dynamic and static expressions differ in intensity. Namely, dynamic expressions are low in intensity as they are still rising, whereas static ones are fully intense. This aspect seems to compensate the facilitation in the discrimination of dynamic over static facial expressions observed in previous studies [4], and even contributed to a *static advantage*. ERP data provided more insight on the processes underlying the behavioral results.

In line with our first alternative hypothesis dynamic expressions elicited greater P1 than the static, indicating differences in the early visual processing between the dynamic and the static. Interestingly the effect was shifted in latency compared with [4] where presentation mode effects appeared in the EPN. It should be noted that differences between dynamic and static stimuli occurred only in the first 150 ms segment after onset. We propose that time pressure may facilitate noticing the early perceptual differences between dynamic and static stimuli.

According to previous studies [16], [17] the P1 amplitude reflects the processing of low-level features and the allocation of visual attention resources. Therefore, the greater P1 observed in the dynamic condition in the present study supports the dynamic advantage in the attention grabbing at very early stages. The processing of dynamic stimuli demand additional visual processing resources to process the more complex information in dynamic compared to static facial expressions (i.e., multiple frames, motion, and expressional change). Moreover, facial movements engage more visual attention because motion captures attention per se [36]. The lack of significant interaction between the presentation and the facial expressions in the P1 indicates the presence of motion effect across all facial expressions, including neutral.

In line with our second hypothesis, results revealed larger N170 amplitude for static than dynamic facial expressions. This could be partly due to a carry-over effect from the previous P1 modulation (larger for dynamic than static). However, carry-over effects cannot account for the effect of facial expressions within each presentation mode. The supplementary analysis on P1 and N170 revealed that the main effect of facial expression was significant only in the static condition, as larger N170 amplitude for angry than neutral facial expressions. This finding is consistent with previous studies revealing the effect of facial expression on the N170 [18], [33], and suggests that angry static faces received enhanced structural encoding. Emotional effects appeared earlier in the present study (N170) compared with [4], where they started with the EPN. It is plausible that the time pressure urged participants to encode and decode the facial expressions very quickly, shifting the differentiation between expressions to an earlier stage.

It is possible that under conditions of time pressure static expressions, starting with a fully developed facial expression, receive improved structural encoding (reflected in the larger N170 for static than dynamic), resulting in better discrimination among facial expressions (reflected in the larger N170 for anger than neutral). This can be seen as form of *static advantage*.

The EPN amplitudes for angry and happy faces were significantly larger than neutral, across presentation modes. This is consistent with previous studies indicating that the EPN reflects the selective processing of emotional stimuli [30], [31]. In contrast with [4], we did not observe a significant difference between dynamic and static conditions in the EPN amplitude. It is possible that the *dynamic advantage*, already present in the earlier stage of the P1 was inhibited during the EPN time window.

In line with [4], dynamic facial expressions elicited greater LPC amplitude than static ones, possibly reflecting that the dynamic condition received more emotional attention.

There are some limitations in the present study. Firstly, stimuli disappeared when participants responded, resulting in jittered presentation times. Since most RTs overlapped the later time window, the LPC data might be confounded with offset potentials. Second, the use of stimuli morphed with computer software limits the ecological validity of our findings. Although spontaneous facial expressions tend to be uniform and reflex-like in appearance [37], natural expressions probably show more variability in the dynamic properties than the ones used here. Additional research using more natural stimuli will be necessary to confirm and extend the results presented here. Third, given the impact of motion, and since emotional expressions come together with particular physical changes, the essence of the effect of facial expressions on ERPs deserves further study. Lastly, the stimuli included faces from different ethnicities, which might impact recognition. It would be valuable to explore the other race effect in this paradigm.

## Conclusions

The present study shows that during the classification of static and dynamic facial expressions, if the goal-driven attention is focused by setting high time pressure, making the participants process the emotional information as soon as possible, this benefits the processing of static relative to dynamic expressions, possibly for its fully developed facial expressions from the onset. Still, we observed some form of dynamic advantage in the P1 and LPC components, reflecting larger early visual attention and better processing of dynamic expressions at later stages. Under time pressure, the stimulus-driven attention enhanced by motion, guiding processing resources to the emotional expressions, might overtax the resources devoted into the task of facial expression discrimination and delay the encoding, as shown in the greater P1 and smaller N170 for dynamic than static condition.
